# Supplementary material for: NMR analysis of the correlation of metabolic changes in blood and cerebrospinal fluid in Alzheimer model male and female mice
Source: PLoS One. 2021 May 10;16(5):e0250568. doi: 10.1371/journal.pone.0250568 (PMC8109765; doi:10.1371/journal.pone.0250568)
Supplement: S2 Fig — Shown are values prior to normalization. (PPTX) [file pone.0250568.s002.pptx]

## Slide 1
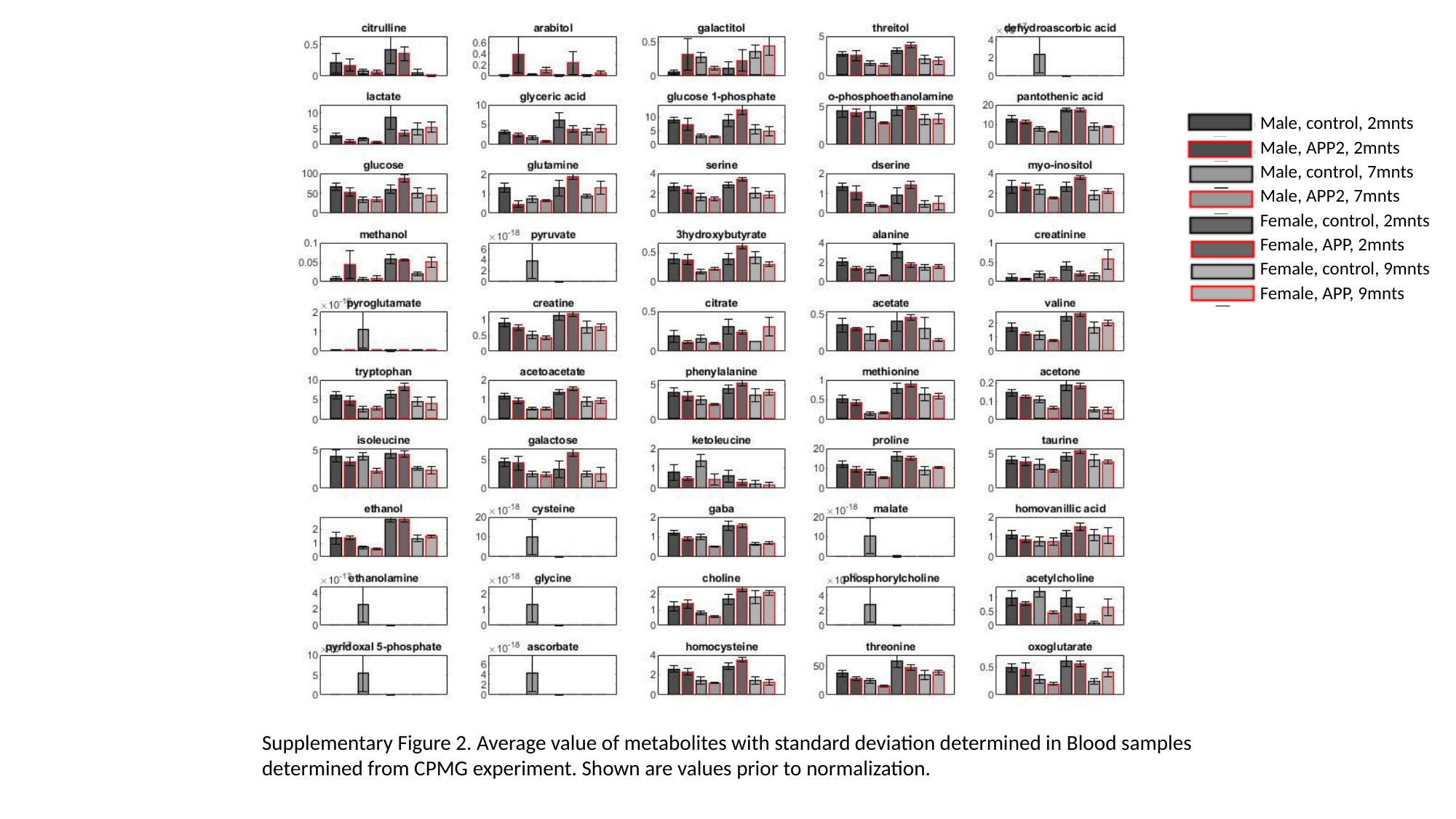

Male, control, 2mnts
Male, APP2, 2mnts
Male, control, 7mnts
Male, APP2, 7mnts
Female, control, 2mnts
Female, APP, 2mnts
Female, control, 9mnts
Female, APP, 9mnts
Supplementary Figure 2. Average value of metabolites with standard deviation determined in Blood samples determined from CPMG experiment. Shown are values prior to normalization.
