# Supplementary material for: NMR analysis of the correlation of metabolic changes in blood and cerebrospinal fluid in Alzheimer model male and female mice
Source: PLoS One. 2021 May 10;16(5):e0250568. doi: 10.1371/journal.pone.0250568 (PMC8109765; doi:10.1371/journal.pone.0250568)
Supplement: Attachment — Submitted filename: reviewer_response.docx [file pone.0250568.s003.docx]

*We would like to thank Editor and the Reviewer for carefully assessing our manuscript and very useful comments and suggestions. We have made all the requested changes and specific answers are shown below. These changes made our manuscript stronger and we are grateful to reviewer for this. Answers to specific questions are shown in Italic.*

Reviewer #1: The Authors present a nice work in NMR analysis on blood and CSF samples in a Alzheimer mice model. A total of 30 samples (90 mice) were prepared and NMR spectra were recorded using NOESY and CPMG pulse sequences. Data were qualitatively and quantitatively analyzed and statistical analysis were performed. 51 metabolite were identified and correlations between metabolites in blood and CSF were determined.

*We would like to thank reviewer for this highly encouraging comment.*

- Authors don’t use buffer to prepare the samples and never give the pH of the samples. For all samples the pH are the same ?

*Following work presented by Canueto et al. (Cañueto, D., Salek, R.M., Correig, X. et al. Improving sample classification by harnessing the potential of 1H-NMR signal chemical shifts. Sci Rep 8, 11886 (2018).) we realized the possibility to utilize chemical shift changes as another metric in assessing the disease effects on body fluid metabolome. Because of that we did not include buffer in the samples. However, our peak position analysis (show through Figure 1) shows now peaks shift that would indicate differences in pH values across samples. Still, in order to correct any possible errors in quantification caused by peak shift we have performed computational peak alignment.*

- Authors indicate that the region “0.5–9.5 ppm were included in the normalization and analysis”, but residual signal of water is present around 4.7 ppm ? Perhaps the information is in the sentence “…, removal of undesired areas, …’ data analysis section, but give more details please.

*Water peak region from 4.5-5.2 ppm was removed (set to 0 intensity) from the analysis. We have added this information to the manuscript.*

- Please give more information about the normalization procedure, it’s not clear.

*Initially all NMR peaks were normalized to the reference peak. Concentrations of metabolites obtained from the spectra were normalized using z-score normalization. This information is added to the manuscript.*

- How many mice the authors have per group ? If it’s only 3, it’s very poor to give reliable results. I have never seem in this paper, the standard deviation on the results, why ?

*In the experiment we used CSF and blood samples from 90 mice. For CSF we had to pool samples for 3 mice in order to get sufficiently strong signal and we had 3 pools (total of 9 mice in total for each category). For blood we had 3 mice for each category. Although it would be of course very beneficial to have a larger pool of mice for this work at this point this was not possible. Additionally, APP2/PS1 mice have a very short life expectancy making this experiment very difficult as it required a larger pool to insure sufficient survival rate. We have now added as supplementary figures bar graphs for all metabolites showing standard variation across samples. This analysis shows only minor variation. As these animals are all kept at exactly the same conditions and are clonal there are no confounding factors other than differences studied here.*

minor correction

- table 1, Column Male/blood, Pantothenic acid and not pantothenicacid

*All metabolite labels have been corrected.*
